# Latent profile categories of fear of falling in older patients with osteoporotic spinal fractures: impact on physical activity at 1 month postsurgery

**DOI:** 10.3389/fpubh.2026.1856164

**Published:** 2026-07-22

**Authors:** Chunshu Wang, Xiong Wang, Xiaofei Wang, Zhouqin Zhao, Zeyan Shuai, Huan You, Tao Liu

**Affiliations:** Luzhou Maternal & Child Health Hospital (Luzhou Second People's Hospital), Luzhou, Sichuan, China

**Keywords:** fear of falling, influencing factors, latent profile, OVCF, physical activity, spinal fragility fractures

## Abstract

**Objective:**

To explore the latent profile categories and influencing factors of fear of falling in patients with osteoporotic vertebral compression fractures (OVCF) after surgery, and to explore the effect of latent categories of fear of falling on physical activity 1 month after surgery.

**Methods:**

A total of 240 patients undergoing surgery for OVCF were enrolled via convenience sampling. The Fear of Falling Questionnaire-Revised was administered to participants on the day of their first postoperative out-of-bed activity. Physical activity levels were subsequently assessed using the Physical Activity Scale for the older adults at the 1-month postoperative follow-up.

**Results:**

There were 228 valid questionnaires (95.00%). Patients’ fear of falling was divided into the high-fear group (28.07%), the medium-fear group (42.11%), and the low-fear group (29.82%). Compared with the low-fear group, the OVCF patients were more likely to develop into the medium level of fear in the past 6 months after surgery (OR = 1.543, *p* = 0.019). Patients with high scores on perceived environmental factors and personal activities, low self-efficacy, and vertebral fracture ≥2 times were more likely to develop into the high-fear type (OR = 1.206, *p* = 0.002; OR = 1.483, *p* < 0.001; OR = 0.886, *p* = 0.015; OR = 1.483, *p* < 0.001; OR = 1.595, *p* < 0.001). There were significant differences in physical activity levels among patients with different profiles after OVCF surgery (*F* = 216.853, *p* < 0.001). Pairwise comparison showed that the physical activity level of the high-fear group was significantly lower than that of the medium-fear group (*p* < 0.001) and the low-fear group (*p* < 0.001), and the physical activity level of the medium-fear group was significantly lower than that of the low-fear group (*p* < 0.001).

**Conclusion:**

Patients with OVCF are more likely to develop a moderate level of fear of falling within the first 6 months after surgery. Patients with high scores in perceived environmental factors and personal activities, low self-efficacy, and a history of vertebral fractures of two or more times are more likely to develop a high level of fear of falling. The management pathway for fear of falling after OVCF surgery should be established with fear stratification, precise intervention, and activity promotion as the core.

## Introduction

Spinal fragility fractures, especially osteoporotic vertebral compression fractures (OVCF), have a high incidence among the older adults. This is an important cause of disability and decreased quality of life in this population (1, 2).

Although surgery can relieve pain and restore vertebral height (3), postoperative patients often experience fear of falling (FOF) or kinesiophobia due to pain (4, 5), decreased balance ability, and concerns about re-fracture. This is manifested as avoidance of daily activities and rehabilitation exercises, seriously affecting the level of physical activity and functional recovery (6). Fear of falling (FoF) is defined as a psychological state characterized by reduced confidence and self-efficacy in performing activities, accompanied by activity avoidance behaviors, driven by persistent worry about experiencing a fall despite having the physical capability to do so (7). This construct can be explained within the framework of the fear-avoidance model, wherein prior fall experiences or impaired balance are perceived as threats, triggering anxiety and low fall-related self-efficacy, which in turn lead to activity avoidance, muscle weakness, and functional decline (8). FoF has been widely reported in various musculoskeletal pain disorders; for instance, patients with fibromyalgia syndrome often exhibit significant FoF due to impaired balance control and widespread pain, which is closely associated with restricted activity. Similarly, patients with inflammatory arthritis, such as rheumatoid arthritis, report FoF prevalence rates ranging from approximately 16% to over 60%, attributed to joint deformities, muscle weakness, and previous fall history. Consequently, FoF represents a critical psychological barrier affecting participation in therapeutic exercise and overall functional prognosis (9, 10).

Epidemiological studies have shown that fear of falling is common among the older adults, particularly in patients with fragility fractures such as OVCF. Fear of falling is not only associated with an increased risk of falling again but also significantly reduces patients’ activity participation and quality of life (11, 12).

Most of the existing studies analyzed fear of falling as a continuous variable, ignoring the possible heterogeneity within it. Latent Profile Analysis (LPA), as a data-driven classification method, can identify internal subgroups (latent categories) with different characteristics based on patients’ response patterns on multiple relevant indicators, so as to more accurately describe patients’ psychological and behavioral characteristics.

Previous studies have applied the latent class growth model to OVCF patients to identify different change latent classes of fear of falling, and confirmed the existence of categories such as “high level with slow decline,” “medium level with rapid decline,” and “low level with continuous stability.” Factors such as advanced age and multiple fractures were found to be associated with a high fear trajectory (13, 14).

However, although existing studies have largely focused on the longitudinal trajectories of fear, few have systematically examined how distinct fear subtypes (classified by multidimensional characteristics) specifically impact early physical activity at a single time point.

On the other hand, studies on the level of postoperative kinesiophobia in patients with spinal fractures show that kinesiophobia is negatively correlated with rehabilitation self-efficacy and compliance, which is an important factor affecting early postoperative activities. However, there is also a lack of refined analysis based on latent categories (15).

In view of this, this study intends to use the latent profile analysis method to classify patients into several latent categories based on fear of falling and related psychological and environmental factors in the early postoperative period of spinal fragility fractures. It will systematically compare the differences in the physical activity levels of different categories at 1 month after surgery, so as to reveal the effect of the latent categories of fear of falling on early physical activity. This aims to provide a theoretical basis and empirical support for the development of stratified and precise rehabilitation intervention strategies.

## Participants and methods

### Participants

From January to December 2025, convenience sampling was used to select OVCF patients treated in the Department of Spinal Orthopedics of Luzhou Maternal & Child Health Hospital as the research subjects.

Inclusion criteria: Imaging diagnosis of fresh thoracolumbar compression fractures; Age ≥ 60 years; Bone mineral density *T* score ≤2.5; Patients who underwent unilateral percutaneous vertebroplasty for the first time; Informed consent and ability to complete the questionnaire through communication.

Exclusion criteria: Pathological fracture; Severe underlying disease or cognitive impairment; Presence of spinal cord injury and neurological symptoms.

A total of 16 independent variables were included, and the sample size was taken as 5 to 10 times the number of variables (16). Considering 20% invalid questionnaires, the required sample size was estimated to be 100–200 cases. To ensure the stability of the study results, the sample size was finally determined to be 240 cases.

This study has been approved by the Ethics Committee of Luzhou Maternal & Child Health Hospital (2025–37, LLSCSQ-1003). All subjects signed the informed consent and voluntarily participated in this study.

### Survey tools

The literature related to the fear of falling in OVCF was retrieved via computer, and the influencing factors were extracted. Chinese databases such as the China National Knowledge Infrastructure, Wanfang Database, VIP database, and China Biology Medicine Disc, as well as foreign language databases such as PubMed, Web of Science, Cochrane Library, and Embase were searched. The search time limit was from the establishment of the database to November 2024.

The Chinese search terms were “spinal fracture/compression fracture/osteoporotic compression fracture,” “fear of falling/falling/fear of falling/fall efficacy/activity avoidance.” The English search terms were “spinal fracture/compression fracture/osteoporotic vertebral compression fracture” and “fear of falling/fall/fall efficacy/activity restriction.”

After literature screening, in combination with expert opinions and discussions by our research team, 15 factors were finally included. Among them, 11 factors and the first time to get out of bed were compiled into a general information questionnaire. Fall risk perception and self-efficacy were investigated using the existing mature scale.

### General information questionnaire

The questionnaire was self-designed by the researchers and included gender, marital status, age, main caregiver, residence, family monthly income, BMI index, history of falls in the past 6 months, number of vertebral fractures, comorbidities, first ambulation time, and pain degree.

### Fear of falling questionnaire-reversed

The Fear of Falling Questionnaire-Reversed (FFQ-R) was established by American scholar Bower et al. on the basis of the revision of the Fall of Fear Scale (17) and was localized by Wang (18). The scale consists of 15 items, which are divided into 4 dimensions: threat degree, future expectations, coping potential, and injury consequences. The Likert 4-point scoring method is used, ranging from “strongly disagree” to “strongly agree” and scoring 1–4 points. The total score ranges from 15 to 60 points, and the higher the score, the higher the level of fear of falling. The content validity index of the Chinese version of FFQ-R ranges from 0.87 to 0.93 at the item level, and the content validity index of the scale is 0.90. The Cronbach’s *α* coefficient of the scale is 0.747, and the split-half reliability is 0.770 (18).

### The fall risk perception questionnaire for patients

The Fall Risk Perception Questionnaire for Patients (FRPQ) was sinicized and revised by Bao Guanjun to evaluate patients’ perception of the uncertainty and severity of fall risk (19), thereby enhancing their initiative in fall prevention. The scale consists of 26 items, encompassing three dimensions: environmental factors, personal activity factors, and physical condition factors. A Likert 4-point scoring method is employed, with scores ranging from 0 to 3 points, from “absolutely no risk” to “extremely high risk.” The total score ranges from 0 to 78 points, and the higher the score, the greater the perceived risk of falling.

The content validity index at the item level of the Chinese version of the FRPQ is 0.860–1.000, the content validity index at the scale level is 0.909, and the Cronbach’s *α* coefficient and split-half reliability of the scale are 0.942 and 0.808, respectively.

### General self-efficacy scale

The General self-efficacy Scale (GSES) was translated into Chinese by Chinese scholar Wang Caikang et al. (20). It is used to evaluate the self-efficacy of patients. The scale consists of 10 items, and the Likert 4-point scoring method is used, ranging from “completely disagree” to “completely agree.” The total score ranges from 10 to 40, and the higher the score, the higher the self-efficacy of the patients. The Cronbach’s *α* coefficient of the Chinese version of GSES is 0.87, the test-retest reliability is 0.83, the split-half reliability is 0.82, and the item-level content validity index ranges from 0.60 to 0.77 (20).

### Physical activity scale for the older adults

The Physical Activity Scale for the older adults (PASE) was developed (21) The scale contains three dimensions, namely leisure activities, housework activities, and work-related activities. The Chinese version was revised by Hong Jun et al., with a total of 12 items. The scoring method is as follows: first, each item is assigned a value according to the frequency, duration, and intensity of the activity, and then all item scores are weighted and summed. Total scores range from 0 to 360, with higher scores indicating a higher level of physical activity. The Chinese version of PASE also showed good reliability and validity in the Chinese older adult population, with a test-retest reliability of 0.90, and had a moderate correlation with the results of objective measurement methods such as accelerometers.

### Methods of data collection and quality control

Before the investigation, the investigators were trained and examined, and the consent of the relevant departments of the hospital was obtained. The Fear of Falling Questionnaire-reversed, the Fall Risk Perception Questionnaire for Patients, and the General self-efficacy Scale were used by the investigators to conduct a questionnaire survey on the first day of ambulation of the patients after OVCF surgery using unified instructions. The Physical Activity Scale for the older adults was obtained at the return visit of the patients 1 month after surgery.

The purpose of the study, the method of filling out the questionnaire, and the principle of confidentiality were explained to the respondents. The patients filled in the questionnaire by themselves after obtaining informed consent. The paper-version of the questionnaire was used. If the patients were old, had a low education level, poor vision, or were inconvenient to fill in the questionnaire, the investigators or main caregivers would help fill in the questionnaire in the form of questions and answers. If the patients had questions, the investigators should maintain an objective attitude to answer them.

The questionnaires were distributed and returned on the spot, and checked and entered by the investigators within 24 h after the questionnaire was returned. For unclear information, the questionnaire was confirmed with the respondents in time. Questionnaires with a response rate of less than 90%, an answer time of less than 2 min, or answer options with obvious rules were excluded.

### Statistical methods

Excel software was used by two people to enter the data, and Mplus 8.3 software was used to analyze the latent profile of the fear of falling of patients after OVCF surgery. Starting from the single-category model, the number of categories was gradually increased, and the model that best fitted the data was found through comprehensive comparison.

Model fitting index:

1. Akaike information criterion (AIC), Bayesian information criterion (BIC). The smaller the BIC and the smaller the aBIC, the better the model fit.
2. The information entropy represents the accuracy of classification, and its value ranges from 0 to 1. The higher the value, the higher the accuracy of classification.
3. The Lo–Mendell–Rubin likelihood ratio test (LMRT) and Bootstrapped likelihood ratio test (BLRT) were used to evaluate the accuracy of classification. With significance *p* < 0.05, it indicates that the model with *k* categories was significantly better than the model with *k*−1 categories.

SPSS 26.0 software was used for data processing and statistical analysis. One-way analysis of variance, chi-square test, or Kruskal–Wallis *H*-test were used for univariate analysis. Multinomial logistic regression analysis was employed to identify the influencing factors of the latent profiles of FoF among postoperative OVCF patients on the day of their first out-of-bed activity. Using Profile 3 (C3) as the reference group, the independent variables were coded as follows: Age (60–69 years = 1, 70–79 years = 2, ≥80 years = 3, reference, 60–69 years); BMI (<18.5 kg/m^2^ = 0, 18.5–23.9 kg/m^2^ = 1, ≥24 kg/m^2^ = 2; reference: <18.5 kg/m^2^); History of falls in the past 6 months (No = 0, Yes = 1; reference: No); History of vertebral fractures (1 = 1, ≥2 = 2, reference, 1); and Pain intensity (Mild = 1, Moderate = 2, Severe = 3; reference: Mild). The significance level was set at *α* = 0.05. The test level α = 0.05.

## Results

### Basic information

A total of 240 questionnaires were distributed. A total of 12 patients were excluded from the group due to having more than 10% of blank items in the questionnaire and refusing to participate in the follow-up (missing the survey content of Physical Activity Scale for the older adults). A total of 228 valid questionnaires were obtained, and the effective recovery rate was 95.00%. The age of patients ranged from 64 to 88 years, with an average of (77.62 ± 2.61) years. Other information is shown in Table 1.

**Table 1:** Univariate analysis of general information and latent profile of fear of falling of the subjects (*n* = 228).

Variables	Overall (*n* = 228)	High fear group (*n* = 64)	Medium fear group (*n* = 96)	Low fear group (*n* = 68)	*χ* ^2^	*p*
Age (years)					11.007	0.026
60 ~ 69	53	8	25	20		
70 ~ 79	113	30	48	35		
≥80	62	26	23	13		
Gender					0.470	0.791
Male	99	30	41	28		
Female	129	34	55	40		
Marital status					1.578	0.454
Married	204	55	86	63		
Divorced/widowed	24	9	10	5		
Primary caregiver					0.728	0.948
Children	120	34	49	37		
Spouse	85	25	36	24		
Others	23	5	11	7		
Monthly household income (yuan)					6.029	0.420
<4,000	57	22	20	15		
4,000 ~ 6,999	87	24	37	26		
7,000 ~ 10,000	53	12	26	15		
>10,000	31	6	13	12		
BMI (kg/m^2^)					10.945	0.027
<18.5	53	21	23	9		
18.5 ~ 23.9	113	32	48	33		
≥24	62	11	25	26		
Have you fallen in the last 6 months					7.612	0.022
Yes	52	22	20	10		
No	176	42	76	58		
Number of vertebral fractures					3.551	0.169
1	200	52	86	62		
≥2	28	12	10	6		
History of vertebral fractures					6.378	0.041
1	193	48	85	60		
≥2	35	16	11	8		
Coexisting chronic diseases					5.637	0.060
Yes	121	42	46	33		
No	107	22	50	35		
First time out of bed					1.423	0.491
Postoperative day 0	135	35	56	44		
Postoperative day 1	93	29	40	24		
Pain Level (NRS)					12.906	0.002
<3	209	52	91	66		
4 ~ 6	19	12	5	2		
≥7	0	0	0	0		

### Scores of fear of falling, risk perception, and self-efficacy of patients after OVCF surgery

#### Latent profile analysis of fear of falling in patients after OVCF surgery

In this study, researchers selected the scores of four dimensions as evaluation indicators for the fear of falling (Table 2). Starting from the benchmark model (with the category number set to 1), a fitting analysis of latent profile models from 1 to 4 was conducted. The specific data are shown in Table 3. Under the condition of ensuring the statistical significance of LRT and BLRT, when comparing the fitting indexes, the smaller the AIC, BIC, and aBIC values, the better the fitting, with the Entropy being ensured to be greater than 0.8. Finally, the three-latent profile models were determined to be the optimal models for the fear of falling in patients after OVCF surgery.

**Table 2:** Scores of fear of falling, risk perception and self-efficacy of patients after OVCF surgery (*n*^−^ = 228, score, *х* ± *ѕ*).

Items	Overall	High Fear group	Medium fear group	Low fear group	*f*	*p*
Total score of fear of falling	34.58 ± 3.55	45.76 ± 3.82	35.04 ± 3.46	23.40 ± 3.56	458.715	< 0.001
Threat level dimension	14.33 ± 2.07	19.43 ± 2.04	14.51 ± 2.14	9.26 ± 2.11	271.334	< 0.001
Future expectations dimension	10.91 ± 1.56	14.51 ± 1.76	10.43 ± 2.02	8.19 ± 1.57	152.680	< 0.001
Coping latent dimensions	4.38 ± 1.30	5.64 ± 1.60	4.55 ± 1.26	2.94 ± 1.25	48.347	< 0.001
Injury consequences dimension	4.97 ± 1.05	6.18 ± 0.92	5.55 ± 1.17	3.01 ± 1.02	115.623	< 0.001
Total score of perceived fall risk	27.56 ± 8.94	38.78 ± 9.34	28.51 ± 8.67	15.66 ± 9.34	72.095	< 0.001
Environmental Factors dimension	11.35 ± 4.37	16.12 ± 6.34	11.80 ± 5.89	6.21 ± 4.02	31.615	< 0.001
Personal activity factor dimension	9.64 ± 3.58	13.58 ± 3.01	9.96 ± 3.41	5.48 ± 4.23	65.219	< 0.001
Physical condition dimension	6.57 ± 2.95	9.08 ± 2.89	6.75 ± 3.17	3.97 ± 3.16	33.822	< 0.001
Total self-efficacy score	24.83 ± 6.99	20.43 ± 5.16	24.63 ± 6.15	29.27 ± 7.04	22.810	< 0.001

**Table 3:** Comparison of fitting parameter indexes of different latent profile models (*n* = 228).

Model	AIC	BIC	aBIC	Entropy	LRT	BLRT	Class probability
1	22943.219	22980.233	22957.847				
2	21317.960	21364.245	21228.738	1.000	0.000	< 0.001	0.35/0.65
3	20407.971	20553.720	20422.864	0.925	0.004	< 0.001	0.28/0.42/0.30
4	21202.532	21471.868	21315.859	0.870	0.096	< 0.001	0.23/0.28/0.24/0.25

#### The average attribution rate of three latent categories of fear of falling in patients after OVCF surgery

To verify the reliability of the results of the three-latent-profile category analysis, the average attribution probability of the three class samples within each class was calculated. The results show that the average attribution probability ranges from 96.8 to 98.2%, all of which are greater than 90%, indicating that the three latent profiles are reliable, as shown in Table 4.

**Table 4:** Average attribution quantity rate of three types of fear of falling (*n* = 228).

Models	C1	C2	C3
C1	0.968	0.020	0.012
C2	0.020	0.974	0.006
C3	0.012	0.006	0.982

#### Latent category characteristics of fear of falling in patients after OVCF surgery

**Figure 1:**
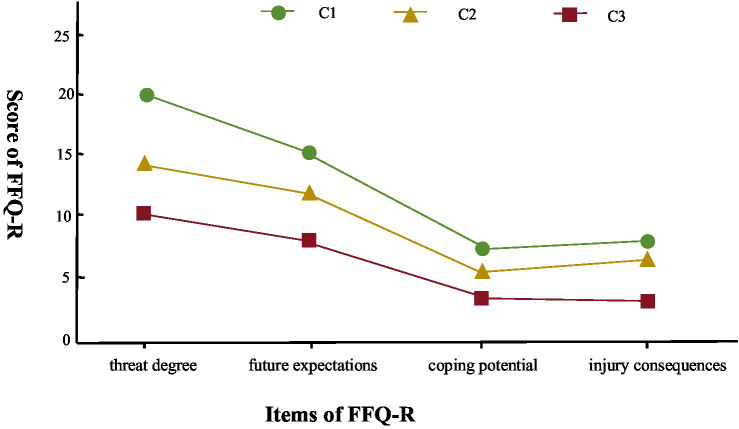
Line chart of latent class profiles of fear of falling among patients after osteoporotic vertebral compression fracture (OVCF) surgery.

Based on the results of the latent profile analysis, we found that patients after OVCF surgery could be divided into three categories. By creating a line chart of the scores for each dimension of the fall-fear scale (see Figure 1), we can more clearly analyze the characteristics of the fear of falling in postoperative OVCF patients in the three latent categories. The three latent categories were named as follows: Category 1 (C1) was the “high-fear group,” accounting for 28.07% (64 cases); Category 2 (C2) was the “medium-fear group,” accounting for 42.11% (96 cases); and Category 3 (C3) was the “low-fear group,” accounting for 29.82% (68 cases).

#### Univariate analysis of latent categories of fear of falling in patients after OVCF surgery

Significant intergroup differences were observed among the three profiles in terms of age (*χ*^2^ = 11.007, *p* = 0.026), BMI (*χ*^2^ = 10.945, *p* = 0.027), fall history within the past 6 months (*χ*^2^ = 7.612, *p* = 0.022), History of vertebral fractures (*χ*^2^ = 6.378, *p* = 0.041), and pain intensity (*χ*^2^ = 12.906, *p* = 0.002), as detailed in Table 1.

#### Logistic regression analysis of latent categories of fear of falling in patients after OVCF surgery

Logistic regression analysis was conducted with the group of fear of falling in patients after OVCF surgery as the dependent variable, and the indicators with statistically significant differences in univariate analysis as the independent variables. The results of this study showed that compared with the low-fear type (C1), the OVCF patients were more likely to develop into the medium-level fear type in the recent 6 months after surgery (OR = 1.543, *p* = 0.019). Patients with high scores of perceived environmental factors and personal activities, low self-efficacy, and vertebral fracture ≥2 times were more likely to develop into the high-fear type (OR = 1.206, *p* = 0.002; OR = 1.483, *p* < 0.001; OR = 0.886, *p* = 0.015; OR = 1.483, *p* < 0.001; OR = 1.595, *p* < 0.001), as shown in Table 5.

**Table 5:** Logistic regression analysis of change trajectory of fear of falling in patients after OVCF surgery (*n* = 228).

Items	*B*	SD	Walds *χ*^2^	*p*-value	*OR*	95% CI
Group C2 vs. group C3						
Constants	1.024	0.421	5.916	0.016	**–**	**–**
Environmental factor dimension	0.112	0.063	3.160	0.082	1.119	0.996 ~ 1.256
Dimensions of personal activity factors	0.143	0.080	3.195	0.079	1.154	0.986 ~ 1.350
Physical condition factor dimension	−0.028	0.112	0.063	0.805	0.972	0.781 ~ 1.211
Total self-efficacy score	−0.064	0.039	2.693	0.124	0.938	0.869 ~ 1.013
Fall in the last 6 months	0.434	0.185	5.503	0.019	1.543	1.074 ~ 2.218
Vertebral fractures ≥2 times	0.245	0.096	6.513	0.010	1.278	1.058 ~ 1.542
Group C1 vs. group C3						
Constants	1.145	0.356	10.345	< 0.001	3.142	1.564 ~ 6.314
Dimensions of environmental factors	0.187	0.066	8.028	0.002	1.206	1.059 ~ 1.372
Personal activity factor dimension	0.394	0.103	14.632	< 0.001	1.483	1.212 ~ 1.815
Physical condition factor dimensions	0.089	0.124	0.515	0.491	1.093	0.857 ~ 1.394
Total self-efficacy score	−0.121	0.049	6.098	0.015	0.886	0.805 ~ 0.975
Fall in the past 6 months	0.220	0.102	4.652	0.032	1.246	1.020 ~ 1.522
Vertebral fracture ≥2 times	0.467	0.137	11.620	< 0.001	1.595	1.220 ~ 2.087

#### Analysis of differences in physical activity among postoperative OVCF patients with different profiles of fear of falling

The physical activity score of 228 OVCF patients 1 month after surgery was (242.36 ± 12.15). A comparative analysis of the physical activity scores of postoperative OVCF patients with different profiles of fear of falling was conducted. The results showed that there were significant differences in the physical activity levels among postoperative OVCF patients with different profiles (*F* = 216.853, *p* < 0.001). Pair-wise comparisons revealed that the high-fear group had significantly lower scores than the medium-fear group (*p* < 0.001) and the low-fear group (*p* < 0.001), and the medium-fear group had significantly lower scores than the low-fear group (*p* < 0.001). See Table 6 for details.

**Table 6:** Analysis of differences in physical activity of patients after OVCF surgery with different fear profiles of falling.

Variables	High Fear group	Medium fear group	Low fear group	*F*	*p*	Compare the results in pairs
Leisure activity dimension	66.47 ± 4.29	81.98 ± 5.49	91.48 ± 6.47	135.924	< 0.001	C1 < C3^***^, C1 < C2^***^, C2 < C3
Household Activities dimension	67.02 ± 4.68	84.55 ± 5.67	93.17 ± 6.88	141.277	< 0.001	C1 < C3^***^, C1 < C2^***^, C2 < C3
Work-related activities dimension	65.15 ± 5.13	81.66 ± 6.49	90.62 ± 7.60	142.391	< 0.001	C1 < C3^***^, C1 < C2^***^, C2 < C3
Total physical activity score	198.64 ± 11.91	248.19 ± 12.67	275.27 ± 14.58	216.853	< 0.001	C1 < C3^***^, C1 < C2^***^, C2 < C3

*** indicates *p* < 0.001.

## Discussion

Tu et al. (22) pointed out that fear of falling is associated with reduced physical activity and rehabilitation compliance, and is correlated with an increased risk of muscle atrophy, functional degradation, and refracture, thus affecting long-term quality of life and prognosis.

The results of this study showed that the score of fear of falling in OVCF patients after surgery was (34.58 ± 3.55), suggesting that this population generally has a moderate degree of fear of falling. However, it has not yet reached the level of fear that completely inhibits daily activities, and as a whole, they still retain a certain degree of functional participation ability. This phenomenon may be related to postoperative pain relief, improvement of vertebral biomechanical stability, and early intermittent rehabilitation intervention. Although most patients have concerns, they do not completely lose their motivation to move.

However, elevated levels of fear may be associated with weakened compliance and intensity of rehabilitation exercise through psychological mechanisms such as strengthened risk perception and decreased self-efficacy, thereby potentially delaying the process of functional recovery (23, 24).

Therefore, in clinical practice, the moderate fear state should be regarded as the key window for reversible intervention. By implementing the safe transformation of the home environment, developing a progressive activity plan, and carrying out cognitive-behavioral therapy, the progression into high fear or kinesiophobia should be effectively mitigated, so as to maximize the level of postoperative physical activity and the quality of functional recovery on the basis of ensuring safety.

This study found that through the analysis of the latent profile, postoperative patients with OVCF fear can be categorized into three latent profiles: high, medium, and low. It shows that the fear of falling in the population is not evenly distributed and there is significant heterogeneity. Moreover, patients within different groups have different characteristics in terms of psychological reaction models and behaviors.

The classification results suggest that in clinical practice, the fear of falling cannot be regarded as a single-dimension continuous variable for unified intervention. Instead, differentiated strategies should be developed for different latent categories.

For the high-fear group, a multidisciplinary psychology-rehabilitation combined intervention should be implemented to strengthen environmental safety and self-efficacy.

The moderate-fear group should be regarded as the key object for monitoring and early intervention. Phased activity training and cognitive behavioral support should be used to prevent their transition into the high-fear group.

For the low-fear group, maintenance guidance and physical activity promotion can be emphasized. This can optimize the postoperative rehabilitation path on the basis of accurate assessment and improve the overall functional recovery level and quality of life.

Compared to the low-fear profile, patients with a fall in the past 6 months had a significantly higher odds of being classified into the moderate-fear profile (OR = 1.543, *p* = 0.019). This finding implies that a single fall incident is associated with a marked increase in fear of falling, potentially driving patients from a low-fear baseline into a moderate-fear state through enhanced environmental alertness and diminished self-efficacy.

Even if the baseline fear level is low, an actual fall experience is also associated with an increase in environmental vigilance and a decrease in self-efficacy, thus leading to membership in a medium-fear state. A potential explanation for this association is that falls, as a negative stressor, are linked to strengthened risk perception and weakened evaluation of one’s own protective ability, prompting patients to report active avoidance of specific actions in daily activities (25, 26).

Secondly, patients with higher scores in the perceived environmental factors of falling were more likely to be classified into the high-fear type (OR = 1.206, *p* = 0.002). This indicates that when patients subjectively identify multiple latent risks of falling in the environment, they tend to remain in a nervous state and limit their range of activities. This is related to the amplification of environmental risks in individual cognition and the formation of a general sense of threat (27, 28). In clinical practice, targeted environmental safety assessment and transformation guidance should be carried out, including optimizing the living layout, adding armrests and anti-slip facilities, and reducing their perception of environmental threat through field exercises (29).

Higher scores in the personal activity dimension were significantly associated with an increased risk of being classified into the high-fear profile (OR = 1.483, *p* < 0.001). This reflects a correlation with a pessimistic judgment of their own mobility, and they often reduce activities due to anticipation-related anxiety, which is largely correlated with previous functional limitation and pain experience (30, 31). In clinical practice, it is necessary to improve their confidence in activities through staged functional training and accumulated successful experience, so as to encourage them to change from fearing to try to actively participating. Patients with low self-efficacy (OR = 0.886, *p* = 0.015) and a vertebral fracture frequency ≥2 (OR = 1.595, *p* < 0.001) also exhibited a higher likelihood of belonging to the high-risk group of high fear. The former are overly dependent on others due to a lack of control, and the latter are vulnerable in cognition due to a history of repeated fractures. Both groups of people need to undergo targeted psychological intervention and strengthen anti-osteoporosis treatment to reduce the risk of re-fracture and alleviate fear (32, 33).

In summary, a hierarchical monitoring and early intervention system should be constructed based on the above-identified risk factors. In postoperative management, psychological factors, environmental optimization, and functional rehabilitation should be simultaneously emphasized to effectively mitigate the escalation of fear and ensure the level of physical activity.

This study found that there were significant differences in the physical activity level of patients with OVCF in different profiles after surgery. The high-fear group was significantly lower than the medium-fear group and the low-fear group, and the medium-fear group was significantly lower than the low-fear group. This result aligns with psychological and behavioral influence mechanism, that is, higher fear levels are associated with increased patient perception of fall risk, decreased self-efficacy, and a tendency to adopt avoidance strategies. Reducing or even giving up rehabilitation exercises and daily activities leads to a decline in physical activity level (34). The high-fear group often prioritizes safety due to excessive worry about re-fracture or loss of balance, and the range and intensity of their activity are significantly limited. Although the moderate-fear group has a partial willingness to participate in activities, the amount of activity is significantly less than that of the low-fear group due to concerns (35, 36).

The clinical implication is that postoperative rehabilitation should be stratified based on the latent categories of fear. For the high-fear group, cognitive-behavioral intervention and gradual exposure training should be prioritized, combined with environmental safety modification to alleviate fear and improve activity compliance. Positive motivation and functional evaluation should be strengthened to consolidate the activity level in the moderate-fear group. For the low-fear group, the focus should be on maintaining and optimizing the quality of activities, and preventing the neglect of risks due to overconfidence. Through accurate identification and intervention, it is possible to maximize the benefits of physical activity and promote functional recovery while ensuring safety.

### Limitations

This study has several limitations that warrant consideration. First, the cross-sectional design inherently limits the ability to infer causality or temporal sequences. While we identified associations between specific factors and fear profiles, we cannot determine whether a history of falls causesheightened fear or if pre-existing high fear predisposespatients to fall (reverse causality). Moreover, the use of convenience sampling at a single center may introduce selection bias, limiting the generalizability of the identified profiles to broader OVCF populations.

Second, unmeasured confounding factors were not controlled for in the analysis. Variables such as pre-existing psychological distress (e.g., anxiety or depression), cognitive status, social support networks, and pre-operative functional levels may act as confounders, potentially influencing both the perception of fear and the engagement in physical activity. The absence of these adjustments means that the observed associations may be overestimated or partially explained by these omitted variables.

Third, there are measurement limitations. The Fear of Falling Questionnaire-Revised (FFQ-R) and the Physical Activity Scale for the older adults (PASE) rely on self-report, which is susceptible to recall bias and social desirability bias. Additionally, the FFQ-R was administered only at the time of first ambulation; therefore, we captured a snapshot of fear rather than its dynamic evolution.

Finally, this study focused on the immediate postoperative period. We did not assess long-term functional recovery or health-related quality of life across the different profiles. Future research should address these gaps by employing longitudinal designs to track functional outcomes and integrating multidimensional factors such as psychological resilience and social support to build comprehensive predictive models.

### Summary

In this study, latent profile analysis was used to classify the fear of falling in patients after osteoporotic vertebral compression fracture (OVCF) surgery into three latent profile categories, namely high, medium, and low-level groups. Compared with the low-fear type, OVCF patients are more likely to develop a medium level of fear within the past 6 months after a fall. Patients with high scores in the perceived environmental factors and personal activities dimensions, low self-efficacy, and ≥2 vertebral fractures are more likely to develop into the high-fear type. The different profiles of the fear of falling are closely related to the physical activity level of patients after surgery.

In clinical practice, a management path for the fear of falling after OVCF surgery should be established, with fear stratification, precise intervention, and activity promotion as the core.

Firstly, a latent profile assessment of patients in the early postoperative period should be carried out to identify the medium-high-level fear group, and the medium-level group should be the focus of monitoring. Family environmental reform should be guided by formulating an environmental safety checklist, such as installing bed fences and removing ground obstacles. This should be combined with step-by-step activity training, gradually transitioning from sitting on the side of the bed to short-distance walking, so as to prevent the progression to high-level fear.

Secondly, the high-fear group should be given multidisciplinary joint intervention. Rehabilitation therapists should conduct environmental risk visualization education and guide patients to record successful experiences to improve their self-efficacy. The orthopedics and endocrinology departments should strengthen anti-osteoporosis treatment to address the history of multiple fractures and introduce low-load activities such as water exercise.

Finally, the fear profile should be dynamically correlated with the level of physical activity. Routine activity prescriptions should be formulated for the low-fear group, and individualized progressive goals should be set for the medium-and high-fear groups. At the same time, environmental reform and safety-accompanying measures should be implemented through the cooperation of doctors, nurses, patients, and their families, so as to effectively reduce fear and maximize the postoperative physical activity participation and rehabilitation quality of patients.

### Public health implications of fear-of-falling profiles in older adults with osteoporotic spinal fractures

From a public health perspective, the identification of heterogeneous fear-of-falling profiles among older adults with osteoporotic spinal fractures highlights an important yet underaddressed component of healthy aging and fall prevention at the population level. Routine fear stratification in community-based elder care and discharge planning could facilitate early detection of at-risk older adults, reduce activity-limiting anxiety, and prevent the cascade from fear to deconditioning, falls, and dependency—outcomes that place substantial burden on families and aged-care systems. Integrating brief fall-fear screening into community health assessments, home-based older adult service programs, and post-fracture follow-up registries would enable targeted public education on environmental safety and self-efficacy enhancement, particularly for frail or recurrent-fracture subgroups. Such a public-health-oriented approach supports active aging, decreases fracture-related disability, and informs policy development for standardized post-fracture psychosocial management in aging populations.

## Data Availability

The original contributions presented in the study are included in the article/supplementary material, further inquiries can be directed to the corresponding author.
